# Editorial: In-depth understanding of post-harvest metabolomic and molecular responses of fresh produce

**DOI:** 10.3389/fpls.2025.1737267

**Published:** 2025-11-19

**Authors:** Natalia Falagán, María E. García-Pastor

**Affiliations:** 1 Plant Science Laboratory, Cranfield University, Bedfordshire, United Kingdom; 2 Department of Applied Biology, Institute for Agri-Food and Agro-Environmental Research and Innovation (CIAGRO), University Miguel Hernández, Alicante, Spain

**Keywords:** fresh produce, post-harvest metabolomic responses, molecular, sustainable technology, food production

## Introduction

Post-harvest is a critical period that significantly influences the quality, storage and shelf life, and nutritional value of fresh produce. Once fruit and vegetables are detached from the plant, they undergo complex biological processes that lead to deterioration, weight loss, and susceptibility to physiological and pathological disorders. Understanding the molecular and metabolic changes during this stage is essential for developing effective strategies to minimise food loss and waste. It is important to note that food loss and waste encompass not only the physical loss of produce but also the decline in nutritional quality that occurs during post-harvest. Preserving the nutritional integrity of fruits and vegetables is therefore vital to ensuring global health.

Recent research has increasingly relied on advanced multiomics analyses, including metabolomics, transcriptomics, and epigenetics (ATAC-seq), to provide a detailed mechanistic understanding of post-harvest physiology. Key regulatory responses involve hormonal signalling, such as those modulated by abscisic acid (ABA), ethylene, and salicylic acid (SA), which control ripening, senescence, and defence mechanisms.

This Research Topic compiles recent advancements in metabolomic and molecular analyses, aiming to provide an in-depth understanding of the mechanisms and responses activated in fresh produce following harvest.

## Overview of research contributions

The manuscripts published in this Research Topic converge on the idea that fruit and vegetable post-harvest life is governed by the interplay between hormonal signalling, oxidative balance, and cell wall metabolism, underpinned by transcriptional and metabolic reprogramming. They differ in crop and stress type but share the goal of understanding and manipulating molecular and structural determinants of post-harvest resilience.

The research conducted by Dobón-Suárez et al. demonstrated that the pre-harvest application (foliar spray vs irrigation) of SA delayed ripening and senescence in green pepper fruit (*Capsicum annuum* L.). Metabolically, SA treatments enhanced the levels of bioactive compounds, including chlorophylls, total phenolics, ascorbic acid, and dehydroascorbic acid, thereby increasing total antioxidant capacity in both hydrophilic and lipophilic phases. This preservation effect was traced to a molecular mechanism involving the upregulation of antioxidant enzyme genes (*Ca*APX, *Ca*POD, *Ca*PAL, *Ca*DHAR2) detected at harvest. While both application methods yielded comparable positive results, irrigation was identified as the most practical and cost-effective for potential agrifood industry application. This study highlights how hormonal regulation through SA application enhances antioxidant metabolism to delay senescence, a mechanism that echoes across several recent investigations into post-harvest resilience.

Building upon the concept of structural and biochemical maintenance during storage, Tan et al. conducted a comparative analysis of red and green *Toona sinensis* buds under cold storage, focusing on cellulose and hemicellulose dynamics. While green buds were nutritionally richer, red buds displayed superior storage resilience and a more stable antioxidant system. The reduced stability of green buds, characterised by severe blackening, elevated cellulase activity, and increased lignification, indicated a critical role for cell wall metabolism in post-harvest integrity. The positive correlation between hemicellulose content and cellulase activity suggested that degradation of these wall components directly contributed to the reduced structural integrity and accelerated aging of green buds. The emphasis on cell wall composition and enzymatic activity bridges this study with broader work linking metabolic reprogramming and hormonal control to structural preservation.

Expanding this view to post-harvest stress signalling, Fugate et al. explored the rapid transcriptomic and metabolic responses of sugarbeet roots (*Beta vulgaris* L.) following severe mechanical wounding. RNA sequencing revealed nearly 5,000 differentially expressed genes within 24 hours, reflecting major metabolic reallocation. The most enriched pathway was plant hormone signal transduction, particularly ethylene and jasmonic acid biosynthesis and signalling. Ethylene showed a more pronounced and sustained response (up to 240-fold increase in *ACO* gene expression), underscoring its dominance in wound response over jasmonic acid. In parallel, genes involved in phenylpropanoid biosynthesis (e.g. *PAL, C4H, 4CL*) and peroxidase activity were upregulated, indicating rapid synthesis of barrier compounds such as suberin and lignin. Together with the previous studies, this work underscores how hormonal cues, especially ethylene, coordinate antioxidant and structural defence mechanisms during post-harvest stress.

Similarly, Guo et al. investigated senescence regulation between a normal tomato cultivar (‘JF308’) and a storable cultivar (‘YS006’) using integrated transcriptomic, metabolomic, and ATAC-seq analyses. The storable cultivar (‘YS006’) exhibited prolonged shelf life and retained higher quality, accumulating more sugars, organic acids, and flavonoids during storage. Mechanistically, the extended stability was linked to phenylpropanoid and carbohydrate metabolism, and reduced expression of cell wall, degrading enzymes (*CesA, PL, EXPA, XTH*). Epigenetic analysis revealed differential chromatin accessibility and upregulation of *bHLH* transcription factors, highlighting transcriptional control as a key determinant of post-harvest resilience. This study reinforces the idea that hormonal and transcriptional regulation converge on cell wall metabolism to maintain firmness and delay senescence.

In the context of non-climacteric fruit, Navarro-Calderón et al. identified ABA as a biomarker of post-harvest resilience in ‘Krissy’ table grapes under cold storage. ABA levels peaked prior to notable physiological changes (respiration increases, organic acid loss, and sugar hydrolysis) signalling the onset of senescence. The spatial distribution of ABA showed higher concentrations in the distal berry region, while the ethylene antagonist 1-methylcyclopropene delayed degradation but increased mould incidence. This indicates that ethylene signalling is not only central to ripening but also essential for activating antifungal defences. Here, the interplay between ABA and ethylene mirrors earlier findings on the hormonal balance underlying post-harvest performance.

Finally, Shi et al., provided structural insight into how pre-harvest calcium chloride (CaCl_2_) treatment reduces cracking in ‘Li Xiu’ grapes by modulating both hormonal and structural pathways. Optimal application during flowering reduced cracking rates up to tenfold and increased firmness by 16.55%. Calcium (Ca) reduced endogenous ABA levels (by 61.26%) and downregulated ABA biosynthesis genes (*VvNCED1, VvBG1*), suppressing cell wall; degrading enzymes such as polygalacturonase and cellulase. Consequently, pectin and cellulose degradation slowed, cell wall thickness increased, and turgor pressure decreased, collectively enhancing mechanical stability. This finding completes the continuum observed across all studies, demonstrating that fine-tuned hormonal and metabolic regulation preserves cellular structure, delays senescence, and enhances post-harvest resilience.

## Mechanism interplay

Post-harvest quality retention is primarily governed by the precise regulation of key phytohormone pathways and cell wall integrity mechanisms. SA pre-harvest treatment provides a viable strategy for stabilising non-climacteric fruit, delaying ripening in green peppers by significantly enhancing the antioxidant system and upregulating defence genes (*CaAPX, CaPOD, CaDHAR2*). Conversely, in table grapes, ABA functions as a senescence biomarker, with a distinct ABA peak preceding critical physiological declines, including increased respiration rate, decreased firmness, and elevated mould incidence. Genetic and molecular approaches reveal that prolonged shelf-life, exemplified by the ‘YS006’ tomato cultivar, stems from the tight control of cell wall metabolism via lower expression of key degrading enzymes (*CesA, PL, EXPA, XTH*), a principle utilised in grapes where Ca treatment reduces cracking by inhibiting ABA synthesis and suppressing cell wall degrading enzyme activities (polygalacturonase and cellulase). Mechanistically, integrated multiomics (transcriptome, metabolome, ATAC-seq) is crucial for identifying the upstream epigenetic regulators, such as specific *bHLH* transcription factors, responsible for modulating these complex stability phenotypes.

The phenylpropanoid pathway emerges consistently as a foundational mechanism for resilience across diverse produce, driving the synthesis of protective phenolic compounds and flavonoids crucial for enhanced antioxidant capacity in SA treated pepper and high-storage tomato cultivars, and initiating rapid wound-sealing barriers (via upregulation of *PAL, C4H, 4CL*) following injury in sugarbeet roots. Hormonal signalling exhibits a nuanced and critical duality: while ethylene dominates the rapid, high-magnitude transcriptional response necessary for wound repair in sugarbeet roots (exceeding jasmonic acid signalling), the same pathway’s inhibition via 1-methylciclopropene in table grapes demonstrates a drawback, delaying sugar degradation but simultaneously compromising the fruit’s defence mechanisms and leading to increased fungal decay risk. Across fruits and vegetables, successful quality retention and firmness maintenance hinge on the molecular regulation of the cell wall structure, requiring either intrinsic genotypic control (tomato) or external chemical intervention (Ca in grapes) to suppress the activity of cell wall degrading enzymes and preserve the structural polymers (cellulose, pectin, hemicellulose), thereby extending post-harvest durability. The integration of advanced analytical techniques and pre-harvest interventions offers promising avenues to enhance the quality and shelf-life of fresh produce.

## Conclusions

These studies emphasise that post-harvest resilience and quality retention in fresh produce are governed by complex and tightly interwoven molecular and metabolic regulatory networks, particularly centred on hormonal signalling (SA, ethylene, ABA) and cell wall metabolism (cellulose, hemicellulose, pectin, lignification). While external interventions (SA and Ca applications) successfully modulate structural integrity and antioxidant defence by controlling key degradation enzymes and hormone levels, endogenous stress responses (wounding) rely heavily on rapid and dominant hormonal cascades (ethylene over jasmonic acid in sugarbeet) to initiate repair. Also, multiomics approaches highlight that superior storage phenotypes in cultivars result from intrinsic differences in the genetic and epigenetic control of these pathways, allowing for a sustained maintenance of cell wall integrity and delayed senescence. The critical role of ABA as a biomarker of senescence across non-climacteric fruit underscores the universal importance of hormonal balance in predicting and regulating post-harvest longevity.

## Future trends

To achieve definitive functional understanding, future research must accelerate the adoption of multi-level omics integration, notably combining ATAC-seq data with transcriptomic and metabolomic analyses to precisely identify the epigenetic control regions and functionally characterise the specific transcription factors that orchestrate senescence and stress response programmes. A major trend involves transitioning toward precision post-harvest interventions, requiring detailed spatial and temporal profiling of endogenous signals, such as the observed compartmentalisation of ABA accumulation (e.g. 3-fold higher in the distal section of grape berries), to develop targeted delivery methods that maximise quality maintenance (e.g. delaying sugar degradation) while circumventing detrimental side effects associated with general treatments (e.g. fungal susceptibility following 1-methylcicloproprene use). Finally, extending species and cultivar-specific mechanistic comparisons, such as investigating the contrasting lignification and cell wall dynamics between red and green *Toona sinensis* buds and confirming the dominance of non-canonical wound signalling cascades, is necessary to tailor effective preservation strategies and inform breeding programmes for improved post-harvest traits.

